# Mania a Potu

**Published:** 1861-01

**Authors:** 


*Mania a Potu*—Is, according to Prof. Warren Stone properly the acute *delirium tremens*: the chronic form of delirium caused by the excessive use of stimulants, the violence of the delirium being, in the former instance, mainly due to alcoholic blood-poisoning, the tremor in the other more to the sudden privation of a long-acustomed stimulant. *Mania a Potu*, often the cause of sudden death, under the names of brain-fever and apoplexy, is generally improperly treated. The free use of opium produces an unfavorable effect upon the nervous system, and tends to check the already diminished secretions. The two forms of the disease require, in treatment, corresponding modifications. The indications are, to establish the secretions, disgorge the system of the alcoholic poison, and to introduce proper nutriment. Calomel in small doses, frequently repeated, until from fifteen to twenty grains are taken, should be followed in eight or ten hours by small and repeated doses of saline medicine. After the stomach and bowels are thus disgorged, milk is universally applicable as a nutriment; the addition of lime-water renders it particularly grateful and soothing to the irritated stomach. In all acute cases, alcoholic stimulants should be withheld, and opium in all forms, prohibited. Where the latter is indicated, which is never the case before the system is thoroughly relieved of the alcohol, equal parts of morphia, and tartrate of antimony, given in small and repeated doses, will soon calm the nervous system and induce sleep, without injury to brain or stomach.—*New Orleans Med. and Surg. Journal.*

Dr. L. P. Gebhard considers delirum tremens as a form of gastritis, only distinguished "by the peculiar nature of its cause." He therefore recommends the application of bloody cups over the whole region of the stomach, with the internal use of opiates. Camphor and opium may be given in conjunction, until sleep is induced; or, better, a mixture of sulphate of morphia, extract of hyoscyamus, and extract of valerian, which has proved of decided advantage. Neither alcohol nor fermented drinks are required.—*Med. and Surg. Reporter.*

The jail-physician of Chicago has found ipecacuanha remarkably successful in delirium tremens. Where a case is not of too long standing, he gives it first as an emetic, and afterwards from fifteen to eighteen grains every other day. Shower-baths and strong beef-tea are used besides, but no alcoholic stimulants.—*Journ. of Mat. Med.*

In the Savanah Hospital very satisfactory results have been obtained with the extract of Indian Hemp, six or eight grains every two or three hours. The tincture is preferable to the extract; a teaspoonful of it taken every two hours, induces sleep rapidly.—*Savannah Journal of Medicine.*

Dr. D. L. Gloninger, of Philadelphia, says: "Nothing will answer every indication as well as lupulin. It may be given *ad libitum* without danger; a tablespoonful, if you please, every hour, until sleep is produced. As much as six pounds of the tincture, made with pure brandy, have been given, before the narcotic effect followed."—*Med. and Surg. Reporter.*

The fluid extract of lady's slipper has been used by Dr. Simms, of Wilmington, Del., with entire success; a tablespoonful every hour, until sleep is produced, which is generally in twelve hours. The remedy is afterwards continued in smaller doses until the patient gets well.—*Journal of Materia Medica.*

